# Reverse shoulder arthroplasty with a 155° neck-shaft angle inlay implant design without reattachment of the subscapularis tendon results in satisfactory functional internal rotation and no instability: a cohort study

**DOI:** 10.1186/s10195-024-00755-5

**Published:** 2024-02-28

**Authors:** Arno A. Macken, Wouter J. van der Poel, Geert A. Buijze, Joris J. Beckers, Denise Eygendaal, Laurent Lafosse, Thibault Lafosse

**Affiliations:** 1 Alps Surgery Institute, 4 Chemin de La Tour de Reine, Clinique Générale d’Annecy, 74000 Annecy, France; 2 Department of Orthopaedics and Sports Medicine, Erasmus Medical Centre, Doctor Molewaterplein 40, 3015 GD Rotterdam, The Netherlands; 3 Department of Orthopaedic Surgery, Amsterdam UMC, Meibergdreef 9, 1105 AZ Amsterdam, The Netherlands; 4 Department of Orthopaedic Surgery, Montpellier University Medical Center, Lapeyronie Hospital, University of Montpellier, 371 Avenue du Doyen Gaston Giraud, 34090 Montpellier, France; 5 Department of Orthopaedics and Traumatology, AZ Sint-Jan Hospital, Mariastraat 38, 8000 Brugge, Belgium; 6 Department of Orthopaedics and Traumatology, AZ Sint-Lucas Hospital, Sint-Lucaslaan 29, 8310 Brugge, Belgium

**Keywords:** Arthroplasty, Replacement, Shoulder, Rotator Cuff, Range of Motion, Articular, Patient Reported Outcome Measures, Joint Instability

## Abstract

**Background:**

The aim of this study was to use the Activities of Daily Living which require Internal Rotation (ADLIR) questionnaire to assess the functional internal rotation in patients who had undergone reverse shoulder arthroplasty (RSA) without reattachment of the subscapularis (SSc) tendon at a minimum follow-up of 2 years. The secondary aim was to report the objective range of motion (ROM) and the rate of postoperative instability.

**Materials and methods:**

All consecutive primary RSA procedures without reattachment of the SSc tendon that were performed using a Delta Xtend prosthesis (an inlay system with a 155° neck-shaft angle) between January 2015 and December 2020 were identified to ensure a minimum follow-up of 2 years. Patients were contacted and requested to fill in several questionnaires, including the ADLIR and Auto-Constant scores.

**Results:**

In total, 210 patients met the inclusion criteria; among those patients, 187 could be contacted and 151 completed questionnaires (response rate: 81%). The SSc tendon was fully detached without repair in all cases, and a superolateral approach was used in 130 (86%) cases. The median follow-up was 4.5 years (range: 2.0–7.6). At final follow-up, the mean ADLIR score was 88/100 (interquartile range (IQR): 81–96). The median level reached in internal rotation was the 3rd lumbar vertebra (IQR: lumbosacral region—12th thoracic vertebra). Of the 210 eligible patients, one required a revision for a dislocation within the first month after primary surgery. With regards to regression analysis with ADLIR score as the outcome, none of the factors were associated with the ADLIR score, although age and smoking approached significance (0.0677 and 0.0594, respectively). None of the explanatory variables were associated with ROM in internal rotation (*p* > 0.05).

**Conclusions:**

This study demonstrates that satisfactory ADLIR scores and internal rotation ROM were obtained at mid-term follow-up after RSA leaving the SSc detached. Leaving the SSc detached also did not lead to high instability rates; only one out of 210 prostheses was revised for dislocation within the first month after primary surgery.

*Level of evidence* III.

## Key points

- This study reports the patient-reported and clinical outcomes of reverse shoulder arthroplasty using a 155° neck-shaft angle inlay implant design without reattachment of the subscapularis tendon.
- Satisfactory patient-reported internal rotation in activities of daily living and range of motion in internal rotation are achieved, along with a low instability rate.
- These results show that leaving the subscapularis tendon detached results in good functional internal rotation and does not cause high rates of instability.

## Introduction

Reverse shoulder arthroplasty (RSA) is an effective surgical treatment option for irreparable rotator cuff tears, cuff tear arthropathy, complex proximal humerus fractures and severe osteoarthritis [21, 25, 35, 42]. Apart from cases of massive non-repairable subscapularis (SSc) tears or cuff tear arthopathy involving the SSc, the SSc tendon is most commonly detached during the surgical approach. After implanting the prosthesis, the tendon can be reattached or be left detached. In some cases, the SSc is torn and not reparable due to fatty infiltration and/or extensive retraction. Potential advantages of reattaching the SSc are increased humeral joint stability and better internal rotation [7, 15, 16, 18, 38]. However, reattachment of the SSc may also impair glenohumeral motion in external rotation, especially in lateralised prostheses. Furthermore, the reattached SSc functions as an adductor after RSA, which increases the force required by the deltoid muscle to elevate the arm [20].

In recent years, some studies have examined the postoperative outcomes of RSA with and without SSc reattachment, reporting no significant differences in postoperative shoulder function between patients who underwent SSc reattachment and patients in which the SSc was not reattached [11, 22, 26, 32, 33, 39, 44]. However, other studies suggest that SSc reattachment is associated with improvement of the postoperative internal rotation of the shoulder [16, 38]. A more recent study highlighted the role played by a healed SSc in improving the range of motion in internal rotation without causing any significant differences in Constant score. However, only half of the SScs had healed in this series [10]. Furthermore, some studies suggest that lateralisation and retroversion may influence the role of the SSc with regards to the outcomes after RSA [33, 40, 47, 48].

Internal rotation is crucial to perform daily activities, especially personal hygiene, which is associated with patient satisfaction after RSA [9]. However, functional internal rotation after primary RSA has been shown to require more complex movements rather than internal rotation alone, such as retropulsion and adduction [24]. Consequently, evaluating internal rotation alone does not seem to be as relevant as evaluating function in internal rotation, which takes into consideration the combination of multiple movements necessary to accomplish a certain task.

In contrast to active internal rotation measured in a clinic, functional internal rotation can be measured using validated patient-reported outcome scores such as the Activities of Daily Living which require Internal Rotation (ADLIR) score [2, 49]. To our knowledge, only three studies on RSA have investigated postoperative internal rotation using patient-reported outcomes for daily activities [1, 2, 36]: two studies investigated RSAs with combined tendon transfer [1, 36] and one pilot study (performed at our centre) that validated the ADLIR questionnaire and reported ADLIR scores 2 years postoperatively after RSA in a small cohort [2].

No large cohort studies have investigated postoperative functional internal rotation after RSA without SSc reattachment. Therefore, this study’s primary aim was to assess the functional internal rotation of patients who had undergone RSA without reattachment of the SSc tendon. The secondary aim was to report the postoperative instability based on the rate of dislocation. We hypothesise that the majority of patients had satisfactory postoperative functional internal rotation at a minimum follow-up of 2 years after RSA without SSc reattachment, with low rates of instability.

## Materials and methods

This is a retrospectively identified cohort study. The protocol for this study was approved by our institution’s regional review board.

### Patient selection

All consecutive primary RSA procedures performed without reattachment of the SSc muscle using a Delta Xtend prosthesis between January 2015 and December 2020 were identified to ensure a minimum follow-up of 2 years. In the case of bilateral arthroplasty, only the first procedure was included to avoid experience bias. Patients who were deceased, non-French speaking or who had no contact information were excluded. Patients eligible for follow-up were contacted by e-mail and telephone. After informed consent was obtained, patients were asked to fill in a patient-reported outcome measure (PROM) questionnaire.

### Surgical technique

All surgeries were performed by two fellowship-trained orthopaedic shoulder surgeons using the same standardised technique. In all cases, a Delta Xtend prosthesis was used, which is standard practice at our institution. The prosthesis is based on the original Grammont design [4] but includes a revised design and a modification concerning the humeral and glenoid components that allows a posterior offset of the humeral component, a high-mobility polyethylene component, and an eccentric glenoid component on a new design of the metaglene. Some parameters were not changed with respect to the original design, such as the 155° humeral neck-shaft angle and non-lateralised glenoid component [41]. The standard preferred surgical technique in our practice is a superolateral approach, which was used unless pre-operative imaging indicated that an inferior extension of the incision might become necessary, most commonly due to an inferior osteophyte, in which case a deltopectoral approach was used. For both approaches, the SSc tendon was either absent or fully detached in all cases regardless of the approach, and the SSc tendon was not reattached. The placement of the humeral component was determined using an intramedullary guide at 20° to 30° of retroversion according to the anatomy of the patient. The metaglene was positioned at the inferior edge of the glenoid. In general, a size 42 glenosphere was used to achieve sufficient inferior overhang, although a size 38 glenosphere was occasionally used in small female patients. Patients were given a sling for 6 weeks after surgery, and early gentle active mobilisation was performed under the guidance of a physiotherapist.

### Implant position on postoperative radiographs

Radiographs taken at 6 weeks postoperatively were assessed to evaluate the position of the prothesis. The follow-up period of 6 weeks was chosen as the initial postoperative radiograph obtained in the recovery room is generally not precise because the radiographs are often taken in bed while there is still significant swelling, which may result in suboptimal positioning of the arm and scapula. After 6 weeks, no changes in position or osseous structures compared to the initial postoperative period—such as bone resorption, ossification or notching—can be expected, resulting in the most accurate radiographic measurements. When no radiograph taken at 6 weeks postoperatively was available, the available radiograph taken closest to 6 weeks postoperatively was used. The lateralisation shoulder angle (LSA) and distalisation shoulder angle (DSA) were measured as described by Boutsiadis et al. [5]. The LSA was defined as the angle between a line drawn from the superior glenoid tubercle to the most lateral border of the acromion and the line drawn from this point to the most lateral border of the greater tuberosity. The DSA was defined as the angle between a line drawn from the most lateral border of the acromion to the superior glenoid tubercle and the line drawn from this point to the most superior border of the greater tuberosity. The sphere-bone overhang distance (SBOD) was measured, as described by Duethman et al. [14], as the distance from a line drawn at the inferolateral edge of the glenoid parallel to the peg to a parallel line at the most inferior aspect of the glenosphere. The measurements were independently performed by two authors. When the difference in angle was less than 5° or the difference in overhang was less than 2 mm, the average of the two measurements was used as the reported outcome. Instead, if the difference between the two measurements exceeded this limit, the disagreement was resolved by re-evaluation, discussion and consensus. The thresholds of 5° and 2 mm were chosen based on the difference that was considered clinically relevant according to the senior authors.

### Outcome variables

All patient characteristics, complication data and revision data were extracted from the patients’ charts and collected for all the patients meeting the inclusion criteria. A revision was defined as any unplanned surgical procedure on the ipsilateral glenohumeral joint. A procedure on the ipsilateral shoulder that was unrelated to the primary arthroplasty, such as an acromioclavicular intervention, was not considered a revision. A complication was defined as any unforeseen medical problem caused by the RSA procedure which negatively influenced the outcome temporarily or permanently.

The following questionnaires were completed: ADLIR, Activities of Daily Living which require External Rotation (ADLER), Subjective Shoulder Value (SSV), Auto-Constant score, American Shoulder and Elbow Surgeons (ASES), and a pain score [8, 19]. In a previous study, the patient-reported Auto-Constant score showed excellent correlation with the clinician-assessed Constant–Murley score [8]. The ADLIR score, as described by Werthel et al. [49], consists of nine questions on activities requiring internal rotation and results in a score ranging from 14 (unable to perform any of the activities) to 100 (no difficulty in performing all activities) (Table 1). A score of 79 or higher, which implies that a patient experiences some or no difficulties in all activities requiring internal rotation, was considered as satisfactory postoperative functional internal rotation. Range of motion (ROM) in internal rotation was extracted from the Auto-Constant (Constant and Murley) score (Table 2). The pain score consisted of Visual Analogue Scales (VASs) for pain during the day, the night and movement, resulting in a total score ranging between 0 (no pain) and 100 (the most severe pain).

**Table 1:** Questions in the Activities of Daily Living which require Internal Rotation questionnaire

Activity	Answer	Score
1. Does your loss of internal rotation affect the global function of the shoulder?	Significantly	6
	Moderately	10
	Occasionally	15
	Not at all	20
2. Is it difficult for you to reach the top of your back with the affected arm? 3. Is it difficult for you to reach your lower back with the affected arm? 4. Is it difficult for you to reach the affected arm for personal hygiene? 5. Is it difficult for you to reach your opposite shoulder and/or axilla with the affected arm? 6. Is it difficult for you to button your shirt? 7. Is it difficult for you to fasten your belt? 8. Is it difficult for you to tie your shoes? 9. Is it difficult for you to open/close a door/curtains?	Impossible	1
	Very difficult	5
	Difficult	6
	Somewhat difficult	8
	Not difficult	10

**Table 2:** Numerical transformation of the internal rotation range of motion using anatomical landmarks

Internal rotation score	
0	Dorsum of hand to lateral thigh
2	Dorsum of hand to buttock
4	Dorsum of hand to lumbosacral region
6	Dorsum of hand to waist (3rd lumbar vertebra)
8	Dorsum of hand to 12th dorsal vertebra
10	Dorsum of hand to interscapular region (7th dorsal vertebra)

### Statistical analysis

Categorical data were represented by numbers and proportions. Histograms and the Shapiro–Wilk test were used to assess distributions for numerical data. Normally distributed data were represented by means and standard deviations and abnormally distributed data by medians and interquartile ranges (IQR). Proportions were represented as percentages with the 95% confidence intervals (CIs). Interobserver agreement in LSA, DSA or SBOD was evaluated using the intraclass correlation coefficient (ICC). The ICC values were interpreted as follows: no agreement to slight agreement, 0.00 to 0.20; fair agreement, 0.21 to 0.40; moderate agreement, 0.41 to 0.60; substantial agreement, 0.61 to 0.80; almost perfect agreement, 0.81 to 1.00 [27].

Three linear regression models were created with the ADLIR score, the internal rotation ROM and the ADLER score as the outcome variable, respectively. All variables displayed in Tables 3, 4 and 5 were included as explanatory variables in the initial model. For each explanatory variable, a linear relation was checked for and adjusted using logarithmic transformation if necessary. Backwards selection based on the* p* value of the correlation between the explanatory and outcome variables was used to arrive at the final model that included only significant explanatory variables. Compliance with the assumptions of linear regression (linearity, independence, normality, homoscedasticity, outliers and multicollinearity) was checked for using diagnostic plots.

**Table 3:** Patient characteristics of the study cohort

Patient characteristic		Patient characteristic	
Female, *n* (%)	95 (63)	ASA score, *n* (%)	
Age, median (IQR)	73 (68–78)	1	42 (28)
Body mass index, median (IQR)	26.4 (23.7–29.0)	2	86 (57)
Diagnosis, *n* (%)		3	22 (15)
Cuff tear arthropathy	78 (52)	Unknown	1 (1)
Osteoarthritis	35 (23)	Comorbidities, *n* (%)	47 (31)
Irreparable cuff tear	26 (17)	Cardiovascular	27 (18)
Humeral fracture	7 (5)	Diabetes	12 (8)
Osteonecrosis	3 (2)	Pulmonary	7 (5)
Inflammatory arthritis	1 (1)	Orthopaedic	6 (4)
Posttraumatic	1 (1)	Psychiatric	6 (4)
Previous surgery, *n* (%)	30 (20)	Renal	5 (3)
Rotator cuff repair	24 (16)	Neurological	3 (2)
Other	6 (4)	Oncological	3 (2)
Smoking, *n* (%)	15 (10)		

*IQR* interquartile range, *ASA* American Society of Anesthesiologists

**Table 4:** Surgery characteristics of the study cohort

Surgery characteristic		Surgery characteristic	
Dominant side,* n* (%)		Glenosphere size, *n* (%)	
Yes	55 (36)	42	116 (77)
No	33 (22)	38	35 (23)
Unknown	63 (42)	Retroversion,* n* (%)	
Approach,* n* (%)		0°	6 (4)
Superolateral	130 (86)	10°	3 (2)
Deltopectoral	21 (14)	20°	12 (8)
BIO-RSA,* n* (%)	31 (21)	30°	129 (85)
Acromioplasty,* n* (%)	124 (82)	40°	1 (1)
LD tendon transfer,* n* (%)	5 (3)		

*BIO* bony increased offset, *RSA* reverse shoulder arthroplsty, *LD* latissimus dorsi

**Table 5:** Results of radiographic measurements

Radiographic measurement	Median (IQR)
Lateralisation shoulder angle (LSA), degrees	78.8 (74.5–84.1)
Distalisation shoulder angle (DSA), degrees	60.2 (52.2–65.8)
Sphere-bone overhang distance (SBOD), mm	4.7 (2.4–6.2)

*IQR* interquartile range, *mm* millimetres

Statistical analysis was performed using R version 4.2.1 (R Foundation for Statistical Computing, Vienna, Austria) and R Studio (R Studio Public Benefit Corporation, Boston, USA).

## Results

**Fig. 1:**
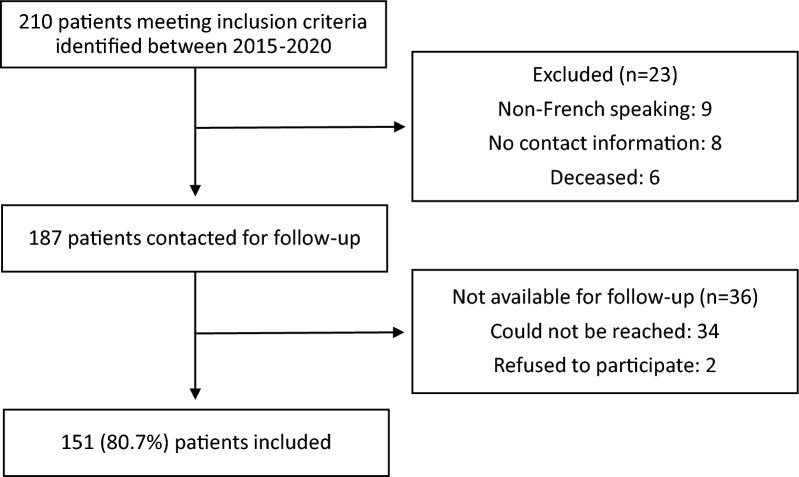
Flowchart for patient selection

Between 2015 and 2020, 210 patients meeting the inclusion criteria were identified. In total, 187 patients were contacted, and 151 patients completed questionnaires (response rate: 81%; Fig. 1) at a median follow-up of 4.5 years (range: 2.0–7.6). The median age at the time of primary RSA was 73 years (IQR: 68–78). The majority of the patients were female (*n* = 95, 63%), and the most common diagnosis for patients who underwent RSA was cuff tear arthropathy (*n* = 78, 52%; Table 3). A superolateral approach was used in 130 (86%) cases. A glenosphere size of 42 (*n* = 116, 77%) and a retroversion of 30° (*n* = 129, 85%) were most common. Bony increased-offset RSA (BIO-RSA) was performed in 31 (21%) cases and a latissimus dorsi (LD) transfer in 5 cases (3%; Table 4).

### Implant position on postoperative radiographs

In total, 123 radiographs (81%) were available for evaluation. The interobserver agreement was substantial (ICC: 0.78; 95% CI 0.70–0.84) for LSA, almost perfect (ICC: 0.89; 95% CI 0.85–0.92) for DSA, and almost perfect (ICC: 0.85; 95% CI 0.79–0.89) for SBOD (Table 5).

### Complications and revisions

Of the 210 patients who met the inclusion criteria, 15 (7%) had a complication. A revision was required in 9 (4%) cases after a median of 6 months (IQR: 1–14; Table 6). Six complications were treated conservatively: two radial nerve injuries, one axillary nerve injury, one ulnar nerve injury, one plexus injury and one superficial infection.

**Table 6:** Characteristics of the revised cases

Case	Revision characteristics					
	Sex	Age at RSA	Reason for revision	Time to revision, months	Procedure	Components revised
1	Male	59	Inferior impingement	7	Revision	Metaglene, glenosphere, PE
2	Male	69	Infection	7	Arthroscopic I&D	None
3	Male	79	Periprosthetic fracture	0	Revision + bone graft	Metaglene, glenosphere, PE
4	Male	75	Infection	1	Two-stage revision	All
5	Male	75	Infection	1	Revision	All
6	Male	72	Infection	6	Revision	All
7	Male	59	Dislocation	0	Revision	PE
8	Male	78	Loosening	53	Two-stage revision	All
9	Male	70	Septic loosening	18	Two-stage revision	All

*RSA* reverse shoulder arthroplasty, *PE* polyethylene, *I&D* irrigation and debridement

### Patient-reported outcome measures

In total, 151 patients completed PROM questionnaires. At the final follow-up, the mean ADLIR score was 88 (IQR: 81–96; Table 7). Among the questions on specific movements (questions 2–9), the median response score was slightly lower for questions 2 and 3 (reaching the upper or lower back; median: 8/10, IQR: 1–10 and 6–10, respectively). For the remaining questions, the median response score was 10/10 (IQR 10–10; Table 8). In 79% (95% CI 73–86%) of the patients, the ADLIR score was 79 (a satisfactory score) or higher. The median internal rotation score was 6 (reaching the 3rd lumbar vertebra with the dorsum of the hand), with an IQR from 4 (lumbosacral region) to 8 (12th thoracic vertebra; Table 7). With regards to regression analysis with ADLIR score as the outcome, none of the factors were associated with the ADLIR score, although age and smoking approached significance (0.0677 and 0.0594, respectively). The regression analysis found older age and smoking to be associated with a lower ADLER score (*β* = − 0.123, *p* = 0.0256 and* β* = − 3.041, *p* = 0.0293, respectively; Table 9), while none of the surgery characteristics (Table 4) or radiographic measurements (Table 5) were significantly associated with the ADLIR or ADLER score (*p* > 0.05). None of the patient characteristics, surgery characteristics or radiographic measurements were associated with ROM in internal rotation (*p* > 0.05).

**Table 7:** Patient-reported outcomes at the final follow-up

Patient-reported outcome at final follow-up	Median (IQR)	Maximum score
ADLIR	88 (81–96)	100
ADLER	30 (28–30)	30
Subjective Shoulder Value	85 (80–95)	100
Auto-Constant	73 (60–87)	100
ASES	90 (75–97)	100
Pain	3 (0–20)	100
Internal rotation score	6 (4–8)	10

*IQR* interquartile range, *ADLIR* Activities of Daily Living which require Internal Rotation, *ADLER* Activities of Daily Living which require External Rotation, *ASES* American Shoulder and Elbow Surgeons

**Table 8:** Outcome scores for each question in the ADLIR questionnaire

Activity	Median score (IQR)
1. Does your loss of internal rotation affect the global function of the shoulder?	15 (10–20)
2. Is it difficult for you to reach the top of your back with the affected arm?	8 (1–10)
3. Is it difficult for you to reach your lower back with the affected arm?	8 (6–10)
4. Is it difficult for you to reach the affected arm for personal hygiene?	10 (10–10)
5. Is it difficult for you to reach your opposite shoulder and/or axilla with the affected arm?	10 (10–10)
6. Is it difficult for you to button your shirt?	10 (10–10)
7. Is it difficult for you to fasten your belt?	10 (10–10)
8. Is it difficult for you to tie your shoes?	10 (10–10)
9. Is it difficult for you to open/close a door/curtains?	10 (10–10)

*ADLIR* Activities of Daily Living which require Internal Rotation

**Table 9:** Linear regression analysis of factors associated with the ADLER score

Factor associated with the ADLER score	Coefficient	Standard error	*t* value	*p* value
Age	− 0.123	0.054	− 2.255	**0.0256**
Smoking	− 3.041	1.381	− 2.201	**0.0293**

All significant p-values (<0.05) in bold

*R*^2^: 0.049,* F*-statistic: 3.667,* p* value: 0.02801

*ADLER* Activities of Daily Living which require External Rotation

## Discussion

The aim of this study was to use the ADLIR score to assess the functional internal rotation after a minimum follow-up of 2 years following RSA without SSc reattachment. The median ADLIR score was 88 (IQR: 81–96) and 79% of the patients had a score of 79 or higher (considered a satisfactory score). Only one patient (0.5%) required a revision for a dislocation. None of the analysed factors were significantly associated with the ADLIR score in the regression model. Overall, these findings demonstrate that satisfactory results in terms of functional internal rotation at a midterm follow-up can be achieved with RSA without SSc reattachment, along with low rates of instability. Considering the importance of internal rotation in daily activities, these results suggest that the patient’s satisfaction and quality of life are not likely to be impacted by a limitation on the internal range of motion after RSA.

### Functional internal rotation

Few previous studies have evaluated postoperative functional internal rotation using a patient-reported score. Beckers et al. reported a mean postoperative ADLIR score of 88 at a minimum of 2 years follow-up in a small cohort who had undergone RSA without SSc reattachment [2]. The outcomes of this pilot study are congruent with the current study. Only two other studies investigated the ADLIR score after RSA. However, they included patients who underwent RSA combined with a tendon transfer, which is not comparable to the current cohort [1, 36]. The results per question in the current study also revealed that patients consider that their loss of internal rotation slightly affects their general shoulder function and that reaching the back poses a slight difficulty for most patients, but other movements and activities generally pose no difficulty for patients. Consequently, in the case of generally positive results, questions 4 to 9 do not seem to contribute to the total result; they would only be discriminative in the case of poorer results. The first question of the questionnaire, which evaluates shoulder function in internal rotation globally, highly influences the final result. The interpretation of this question may also prove difficult for some patients. Therefore, developing a more specific questionnaire that includes more detailed questions could be considered.

To our knowledge, no previous study has compared the postoperative functional internal rotation between reattachment and no reattachment of the SSc during RSA. However, several studies have compared the clinical ROM between the two techniques. The reported results are contradictory; some studies reported no significant difference between the two groups [11, 22, 26, 32, 39], while other studies suggest that leaving the SSc tendon detached in RSA leads to a loss of internal rotation [16, 38]. Engel et al. described a loss of 8° in ROM in internal rotation when the SSc tendon was not repaired compared to when the SSc tendon was repaired in a small randomised cohort of 50 patients with a follow-up of 1 year [16]. Similarly, Rohman et al. identified that not repairing the SSc was a risk factor for a loss of internal rotation in a large cohort of patients [38]. However, a logistic regression was used for loss of and increase in internal rotation, and no direct comparison of internal rotation between patient groups with and without SSc repair is reported. The exact role of the SSc muscle after RSA remains disputed. Although no comparison could be made between SSc reattachment and no reattachment in the current study, we report a median level of internal rotation reaching the 3rd lumbar vertebra. This result is similar to previous studies reporting internal rotation using anatomical landmarks, regardless of the handling of the SSc. Two studies reported the internal rotation of the entire cohort without making a distinction based on the handling of the SSc. Rohman et al. reported that the mean level reached was lumbar vertebrae 4–5, and Rol et al. reported that the lumbosacral region was the mean level reached [38, 39]. These findings suggest that the postoperative internal rotation in our cohort may be equal or superior to the overall outcomes in the literature, regardless of SSc reattachment.

The majority of the humeral components (85%) were placed in 30° of retroversion﻿. One biomechanical study reported a significant decrease in ROM in internal rotation with greater retroversion (*p* < 0.05) [3]. In contrast, a retrospective study comparing RSA placed in 20° of retroversion with 0° found no difference in ROM or difficulty in activities of daily living with the exception of difficulty in washing the back and fastening a bra behind the back, which was more difficult with 20° of retroversion (*p* = 0.026) [37]. A similar retrospective study found no difference in ROM, strength, Constant score or Oxford score when comparing 0° and 20° of retroversion [12]. Degrees of retroversion was not significantly associated with ADLIR or ROM (outcomes) in the regression models. However, the majority of the components were placed in 30° of retroversion, resulting in a homogeneous cohort that was not suitable for a comparison between different grades of retroversion. Our results show that the internal rotation is satisfactory when the humeral component is placed in 30° of retroversion in most cases. A recent computer-assisted study by Hochreiter et al. reported the ideal component placements in order to obtain the best range of motion in internal rotation; the largest impingement-free functional internal rotation was achieved when combining a posteroinferior baseplate position, a greater inferior glenosphere overhang, a baseplate inclination angle of 90°, 6 mm of glenosphere lateralisation with respect to the baseline setup, a lower NSA and anteversion of the humeral component [23].

In the current study, with regards to regression analysis with ADLIR score as the outcome, none of the factors were associated with the ADLIR score, although age and smoking approached significance (0.0677 and 0.0594, respectively). 

### Stability

Another commonly voiced concern of leaving the SSc tendon detached is humeral joint instability. Edwards et al. compared 62 patients with a reparable SSc to 76 patients with an irreparable SSc [15]. All seven of the postoperative dislocations occurred in the group in which the SSc was irreparable. The authors suggest that an attempt to repair the SSc should be made in every case. However, in this study, the non-SSc-repair population consisted of complex cases in which the SSc was irreparable, resulting in a selection bias. Dislocations are more likely in patients with complex diagnoses, including proximal humeral non-union, fixed glenohumeral dislocation and failed prior arthroplasty. In contrast, in our study, only one dislocation occurred among the 210 patients meeting the inclusion criteria. The dislocation occurred 28 days after surgery. The diagnosis for the primary RSA was osteonecrosis of the proximal humeral head after plate fixation 2 years earlier. The patient underwent revision surgery with replacement of the polyethylene insert. To our knowledge, two meta-analyses explored SSc status in RSA as risk factor for dislocation. One study reported a higher dislocation risk in the case of SSc deficiency. The odds ratio for dislocation was 18.43 (*p* = 0.0006) [29]. The other study explored the influence of SSc repair on dislocation. This study showed no significant difference in risk of dislocation between repair and no repair of the SSc. The dislocation rate was 1.6% in patients with no repair of the SSC and 0.8% in patients with a repair (OR [95% CI] − 0.70 [− 1.82, 0.41] [13]. The differences between the dislocation rates in the literature could be attributed to the use of a different prothesis design or different surgical techniques, along with the placement of the humeral component for which the retroversion was above 20° in 93% of the cases in our cohort. Although humeral component retroversion is associated with decreased stability of the prosthesis in biomechanical studies [17], the high degree of humeral component retroversion in our cohort did not seem to translate to increased dislocation rates. Some other factors could have influenced the observed outcomes, such as the degree of glenoid version, the follow-up time, or the postoperative protocol. In our cohort it was not statistically feasible to analyse the influences of these factors in detail due to the low number of dislocations.

### External rotation

Reattachment of the SSc has been suggested to restrict the ROM in external rotation [40]. The median ADLER score in our study was 30, showing that there was excellent functional external rotation in patients who had undergone RSA without SSc reattachment. To our knowledge, there are no studies investigating the role of the SSc in RSA that report the ADLER score as an outcome. Two studies investigating RSA combined with a tendon transfer reported the ADLER score [34, 50], but those are not comparable to our cohort, who underwent RSA alone. Nonetheless, the excellent ADLER score in our cohort may support the suggestion that the absence of the SSc tendon leads to a less restricted functional external rotation. However, this must be confirmed in a comparative study. Older age at the time of surgery and smoking were associated with a lower ADLER score in the regression analysis (Table 9). Previous studies did not find this correlation. One study comparing cohorts of younger (< 65 years) and older (> 70 years) patients found no difference in PROMs after RSA but a greater range of motion in the younger group [28]. In contrast, one study found higher ASES scores and greater internal rotation ROM in patients older than 60 years compared to younger patients [6]. The median age in the current cohort is notably higher (73 years), potentially explaining the incongruency. In contrast to our findings, one previous study found no difference in PROMs or ROM outcomes between smokers and non-smokers [46]. However, smoking may also be correlated with other factors not measured in the current study, such as socio-economic status, which is associated with worse outcomes [45].

### Radiographic measurements

Boutsiadis et al. established the LSA and DSA as reproducible measurements to estimate lateralisation and distalisation after RSA, and they showed an interobserver agreement of 0.78 (substantial agreement) for LSA and 0.81 (almost perfect agreement) for DSA [5]. Our study showed similar results regarding interobserver agreement; 0.78 (substantial agreement) for LSA and 0.89 (almost perfect agreement) for DSA, confirming that the measurement of these angles is reliably reproducible. Thupé et al. showed lower interobserver agreement in patients who underwent RSA after a proximal humeral fracture; they report fair agreement for LSA and moderate agreement for DSA [43]. However, the authors attributed the lower agreement to the difficulty in analysing tuberosity position in patients who underwent RSA after proximal humeral fracture. Notably, the radiographic measurements (LSA, DSA and SBOD) were not significantly correlated with ADLIR or ROM results in the regression models, suggesting that the placement of the prosthesis, such as the baseplate positioning, amount of reaming and positioning of the humeral component, does not influence the (functional) internal rotation. However, this finding may also be attributed to the cohort size and homogeneity. It is possible that more significant associations may be found in a larger, more heterogeneous cohort. Furthermore, implant positioning is best assessed on computed tomography rather than plain radiographs.

### Limitations

This study has several limitations to consider when interpreting the results. First, the patients were identified retrospectively. Since leaving the SSc tendon detached is standard practice at our centre, it was not possible to compare SSc reattachment with no reattachment. Preoperative assessment of the SSc was also not reported in our series. However, we were able to report detailed and satisfactory results of our technique, which can be compared with current and future literature. Other techniques that were used in a portion of the cases, such as BIO-RSA and tendon transfers, may also have influenced the outcome. Secondly, no preoperative scores were available, so we were not able to compare the postoperative to the preoperative outcomes. Third, in this study we used a single prosthesis design, with a 155° humeral neck-shaft angle and an inlay design. For the glenoid component, an inferior overhang of at least 5 mm is systematically created to avoid impingement, which leads to instability and notching. In the paragraphs above, we compare our results with other studies for which these parameters may be different. The single-prosthesis design and single technique used limit the generalisability of our outcomes but they do increase the internal validity of the study. Fourth, the radiographic measurements are dependent on the position of the arm and the angle at which the radiograph was taken. This may potentially introduce bias due to differences in the resting arm position between patients based on sex or BMI. However, the high interobserver agreement demonstrates the high reliability of the radiographic measurements. Furthermore, the position of the scapula on the thorax and the degree of lordosis were not taken into account, which may also have influenced the ROM in internal rotation [30, 31]. Additionally, the interpretation of the ADLIR questionnaire can differ between patients, potentially introducing a bias. For example, personal preferences and cultural differences may influence the movements required for daily activities such as personal hygiene. Lastly, the questionnaires were administered over the phone or by email, potentially introducing a bias based on the medium that was used. However, using several media to collect data resulted in a response rate of more than 80%, which allowed for the analysis of a large cohort and reduces the chance of bias due to non-responders or missing data.

### Future perspective

Future prospective studies comparing the functional internal and external rotation in daily life between RSA cases with and without SSc reattachment are required to demonstrate the functional superiority of one of the two techniques. Furthermore, studies could compare outcomes between different prosthetic designs and surgical approaches. Longitudinal assessments with preoperative and postoperative scores may provide a more comprehensive understanding of patient outcomes.

## Conclusion

This study demonstrates that satisfactory functional internal rotation in daily life was obtained at mid-term follow-up after RSA leaving the SSc detached. Leaving the SSc detached also did not lead to high rates of instability; of the nine prostheses that required a revision, only one was indicated for a dislocation. These results suggest that the altered biomechanical functioning after RSA may render the SSc obsolete, at least with the currently studied implant (the Delta Xtend prosthesis using a size 42 glenosphere) and positioning technique (retroversion of more than 20°).

## Data Availability

The datasets used and/or analysed during the current study are available from the corresponding author on reasonable request.

## Abbreviations

ADLER: Activities of Daily Living which require External Rotation
ADLIR: Activities of Daily Living which require Internal Rotation
ASES: American Shoulder and Elbow Surgeons
BIO-RSA: Bony increased-offset reversed shoulder arthroplasty
BMI: Body mass index
CI: Confidence interval
DSA: Distalisation shoulder angle
ICC: Interclass correlation coefficient
IQR: Interquartile range
LD: Latissimus dorsi
LSA: Lateralisation shoulder angle
NSA: Neck-shaft angle
PROM: Patient-reported outcome measure
RSA: Reverse shoulder arthroplasty
ROM: Range of motion
SBOD: Sphere-bone overhang distance
SSc: Subscapularis
SSV: Subjective shoulder value
VAS: Visual analogue scale
